# The Device–Object Pairing Problem: Matching IoT Devices with Video Objects in a Multi-Camera Environment

**DOI:** 10.3390/s21165518

**Published:** 2021-08-17

**Authors:** Kit-Lun Tong, Kun-Ru Wu, Yu-Chee Tseng

**Affiliations:** 1 School of Computing Sciences, University of East Anglia, Norwich NR4 7TJ, UK; k.tong@uea.ac.uk; 2 Department of Computer Science, National Yang Ming Chiao Tung University, Hsinchu 30010, Taiwan; 3 College of AI, National Yang Ming Chiao Tung University, Hsinchu 30010, Taiwan; yctseng@cs.nctu.edu.tw; 4 Academia Sinica, Taipei 11529, Taiwan; 5 College of Health Sciences, Kaohsiung Medical University, Kaohsiung 80708, Taiwan

**Keywords:** device–object pairing, IoT, computer vision, data fusion, surveillance

## Abstract

IoT technologies enable millions of devices to transmit their sensor data to the external world. The *device–object pairing problem* arises when a group of Internet of Things is concurrently tracked by cameras and sensors. While cameras view these things as visual “objects”, these things which are equipped with “sensing devices” also continuously report their status. The challenge is that when visualizing these things on videos, their status needs to be placed properly on the screen. This requires correctly pairing visual objects with their sensing devices. There are many real-life examples. Recognizing a vehicle in videos does not imply that we can read its pedometer and fuel meter inside. Recognizing a pet on screen does not mean that we can correctly read its necklace data. In more critical ICU environments, visualizing all patients and showing their physiological signals on screen would greatly relieve nurses’ burdens. The barrier behind this is that the camera may see an object but not be able to see its carried device, not to mention its sensor readings. This paper addresses the device–object pairing problem and presents a multi-camera, multi-IoT device system that enables visualizing a group of people together with their wearable devices’ data and demonstrating the ability to recover the missing bounding box.

## 1. Introduction

The advance of IoT technologies enables millions of devices to transmit their sensor data to the external world. On the other hand, empowered by deep learning, today’s computer vision has significantly improved its object recognition capability. When an IoT device is placed on an object, we intend to not only recognize the object in videos but also recognize the IoT device bundled with the object.

Surveillance systems are widely used in homes, buildings, and factories. However, when abnormal events occur, it usually takes a lot of human effort to check the surveillance videos. With the advance of AI technologies, automatically analyzing video content becomes feasible. RetinaNet [1] and You Only Look Once (YOLO) [2] can identify a variety of objects with high accuracy and efficiency. OpenPose [3] and Regional Multi-person Pose Estimation (RMPE) [4] can perform human pose recognition without using depth cameras or ToF (time-of-flight) sensors.

In most surveillance and security applications, the central issue is to capture abnormal people, objects, and events in the environment. This work studies the *device–object pairing problem* in surveillance videos. Previous and common practices include, but are not limited to, barcode, Radio Frequency ID (RFID), and biometric sensing (e.g., fingerprint and iris recognition). However, these methods require keeping at a very short distance to devices. On the other hand, facial recognition relies on obtaining a face database and does not work under larger shooting angles and occlusions. Furthermore, there are privacy concerns in public domains. Another way to identify persons is to exploit IoT devices, such as smartphones and smartwatches, which have become virtually the users’ IDs. Personal devices can also store owners’ profiles and be used in sensitive domains such as factories, hospitals, and restricted areas [5,6]. Further, with the global deployment of 5G, IoT devices nowadays can communicate almost anywhere. When tracking a group of people by camera, it would be nice to also know their purchase histories and preferable social network tools on their smartphone. This motivates us to study combining computer vision and IoT devices under multi-camera environments.

**Figure 1:**
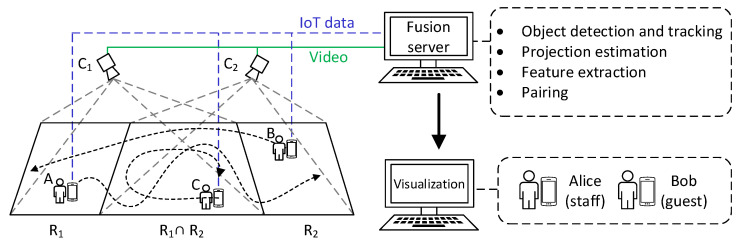
The device–object pairing system architecture.

The device–object pairing system considers an environment consisting of multiple cameras, with users wearing their IoT devices walking around. Figure 1 shows our system scenario. There is an IoT network for collecting data from cameras and wearable IoT devices in the environment. We use a YOLO module to obtain and track the human objects appearing in the cameras. The homography matrices of these cameras are estimated to transform each camera view to common surface space. Because there are multiple cameras that concurrently capture the same set of people, the possibilities of occlusions and tracking failures are reduced. Our system then tries to extract objects’ motion features from both visual and wearable sensors and generates (devices, object) pairs that can be displayed on video objects. Therefore, with IoT devices serving as the personal identification of users, our system can visualize user identities and sensor data for smart surveillance.

We have built a prototype system that runs at 12 Frames Per Second (FPS). It contains a server and two cameras and wearable devices connected by Wi-Fi networks. We designed three mobility patterns (namely random, following, and mimicking) to evaluate its performance.

This paper is organized as follows. Related works are reviewed in Section 2. The proposed system architecture is presented in Section 3. Prototyping and evaluation results are in Section 4. Conclusions are drawn in Section 5.

## 2. Related Work

Surveillance has been a critical issue in smart city for decades. The study in [7] is an early work that shows how a camera with an embedded system and network connection works for smart analysis. Reference [8] proposes sensor-based surveillance with interactive multiple models to track targets. To solve the person re-identification problem, Reference [9] designs a temporal residual learning module to learn the feature of pedestrian sequences. To monitor illegal or suspicious activities, extracting skeletons by deep learning from video frames is studied in [10]. Our research work is more extensive because we consider both video and sensor data.

Human detection technologies have been rapidly developed recently. Reference [11] proposes detecting objects by a Haar Feature-based Cascade Classifier. With the advance of artificial intelligence, References [1,2] achieve real-time detection by deep learning. Skeletons can be extracted from a mono camera frame in [3,4]. For Multiple Object Tracking (MOT), Reference [12] proposes a *k*-shortest paths algorithm, Reference [13] adopts a Kalman filter, and [14] uses a deep learning approach. Multi-camera, multi-person tracking by homography matrices is studied in [15]. In the city-scale environment, reference [16] addresses vehicles re-identification and tracking under a multi-camera scenario. Another multi-camera pedestrian tracking is proposed in [17], in which the IoT devices are edge nodes to analyze video by a deep learning module. A sensor-based IoT architecture for smart surveillance is proposed in [18]. A wireless video surveillance system is presented in [19]. On the contrary, our work explores both wearable devices (with sensors) and cameras.

Sensor fusion-based tracking has been studied in a simpler environment. Reference [20] uses a depth camera to extract skeletons and pair them with the Inertial Measurement Unit (IMU) devices carried by users. Fusion-based human and object tracking is shown in [21]. In [22], they use a camera to capture human motion by OpenPose [3] and match the motion with an IMU device to address ID association. These works all exploit user movements to identify persons. Nevertheless, their detection ranges are limited since skeletons can only be detected within a short distance. Reference [23] addresses the fusion of hand gestures and wearable sensors to identify people in crowded mingle scenarios, but it still requires a short distance to track the movements. Reference [24] is an example using a Received Signal Strength Indicator (RSSI) of Wi-Fi as signal trajectories to pair a person on camera. However, the system is unstable since the RSSI signal may suffer more interference and the variance is large. Reference [25] demonstrates how to tag personal ID on people from a drone camera through trajectory pairing. However, it is limited to one drone. Fusing computer vision and the 5G network for driving safety is proposed in [26]. A reconfigurable platform for data fusion is presented in [27].

In this work, we develop machine learning-based feature extraction and a more efficient fusion model with time synchronization under multi-camera environments.

## 3. Fusion-Based Device–Object Pairing

**Figure 2:**
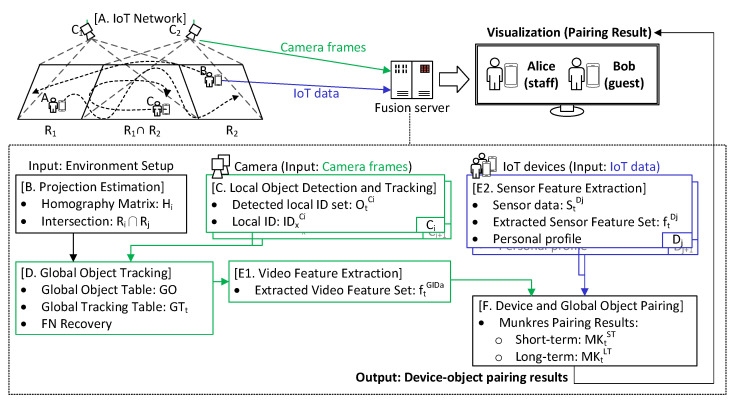
Data fusion procedure.

Figure 2 shows the proposed data fusion process. The hardware components include multiple surveillance cameras, some smart wearable devices with built-in IMU (accelerometer and magnetometer), a fusion server, and an IoT network. To pair devices with video objects, the data collected from cameras and wearable devices will be fused based on features extracted. The main software components include: (A) IoT network, (B) projection estimation, (C) local object detection and tracking, (D) global object tracking, (E) feature extraction, and (F) device and global object pairing. Section 3.1 introduces our IoT network. How to map camera views to a common ground space is addressed in Section 3.2. The object tracking task for each camera is discussed in Section 3.3. How to merge all cameras’ views to a global view is addressed in Section 3.4. Then, feature extraction and device–object pairing are covered in Section 3.5 and Section 3.6, respectively.

### 3.1. IoT Network

In our system, multiple cameras are deployed to fully cover the surveillance site. Users appearing in front of cameras are expected to put on their wearable devices (otherwise, such users will be marked as “unknown” by the pairing module). An IoT network is designed to manage these devices. Data are exchanged by MQTT (Message Queuing Telemetry Transport [28]), which is a lightweight, publish–subscribe protocol allowing message transportation among massive devices without obvious latency. Furthermore, NTP (Network Time Protocol) is adopted to synchronize time among all components.

For convenience, we use smartphones to simulate wearable devices. Each smartphone has IMUs, including a three-axis accelerometer and magnetometer. These sensors periodically report their data to the fusion server at a rate of 50 Hz in JSON format [29]. Readings are marked by timestamps and pre-processed by a low pass filter. Furthermore, each device is uniquely distinguishable by its ID.

There are multiple cameras. For each camera, an M-JPEG (Motion Joint Photographic Experts Group) server is set up for streaming frames by HTTP (Hypertext Transfer Protocol) in the JPEG format. To validate our framework, we do not use the keyframe method [30], where complete frames are interleaved by compressed frames, causing broken textures when a frame is lost. Therefore, all frames are complete frames, and no retransmission is performed. Every frame from camera $C_{i}$ is assigned a timestamp *t* and is denoted as $F_{t}^{C_{i}}$. Frames are remapped to solve the radial distortion problem caused by lens, which is achieved by using the chessboard photos test [31] to calculate the internal and external parameters and the lens distortion coefficients.

### 3.2. Projection Estimation

To relate the views among multiple cameras, we shall map the pixel space of each camera to a common ground space. Our approach is to estimate a homography matrix $H_{i}$ for each camera $C_{i}$ that transforms each camera pixel to a ground coordination. Let the errors $E_{i}$ caused by the transformation follow a normal distribution model and the coverage region of $C_{i}$ be $R_{i}$. We show how to determine (i) $H_{i}$, (ii) $E_{i}$ and (iii) $R_{i}\cap R_{j}$ for each pair of $C_{i}$ and $C_{j}$.

To find $H_{i}$, we design a lightweight human-assisted process. We place only a few markers on the ground and send a designated person to walk in the field arbitrarily. The person passes these markers from time to time. Whenever passing a marker, they will stop for a while and record the measured value before moving forward. This stopping behavior serves as an indication to cameras that they are right on a marker. In addition, another wearable device is attached to trace them. In some sense, the cameras localize the person when they stop at a marker, and the wearable sensor tracks their trajectory between two markers while walking. By calibrating their location to a marker, the drifting problem of IMU tracing is significantly relieved. This procedure can be repeated arbitrarily for all camera coverage regions, and during the procedure, the person’s ground trajectory is mapped with camera views to learn the mapping between pixels and ground trajectories. Lots of such mappings can be found only within a few minutes.

In fact, the above steps serve as a labeling process to map $C_{i}$’s pixel space to the ground space. Through object detection, the center of a human object is regarded as their location in $C_{i}$’s pixel space. Their trajectory in $C_{i}$ is then partitioned into segments (called v_segments) according to those stopping points (markers). On the IMU side, their trajectory is also partitioned into segments (called i_segments) at places where $C_{i}$ reports stopping events. An i_segment is modeled by the recursion: $L_{k}=L_{k-1}+\vec{d_{t}}*stride\_len$, where $L_{0}$ is the location of the starting marker, $L_{k}$ is their location at the *k*-th stride, $\vec{d_{k}}$ is the normalized vector of the *k*-th stride (obtained from magnetometer), and stride_len is a predefined value. The recursion stops when encountering the next marker. We then match the endpoints of all i_segments to ground markers by the maximal likelihood of inter-marker distances and absolute i_segment distances. This derives the ground markers of all i_segments’ endpoints. With known endpoints of each i_segment, we calibrate it by rescaling. Therefore, we obtain many (v_segment,i_segment) pairs for each camera $C_{i}$ with their pixel locations labeled by the i_segment.

Given a large number of (v_segment,i_segment) pairs for $C_{i}$, we can derive its homography matrix $H_{i}$ for the projection. By [32], the objective is to meet

$$\left[\begin{array}{c} u^{\prime }\\ v^{\prime }\\ 1 \end{array}\right]=\left[\begin{array}{ccc} h_{0} & h_{1} & h_{2}\\ h_{3} & h_{4} & h_{5}\\ h_{6} & h_{7} & h_{8} \end{array}\right]\left[\begin{array}{c} u\\ v\\ 1 \end{array}\right]=H_{i}\left[\begin{array}{c} u\\ v\\ 1 \end{array}\right],$$

where (u,v) is a pixel and $\left(u^{\prime },v^{\prime }\right)$ a ground point. It requires at least four pairs to solve $H_{i}$. As an i_segment is more likely to contain errors due to sensor data drifting, we suggest retrieving more knowns to minimize the drifting problem. We then apply the least-square method to find $H_{i}$.

Assuming the projection error $E_{i}$ of $H_{i}$ following a normal distribution, $E_{i}∽N\left(\mu_{i},\sigma_{i}^{2}\right)$, we collect all $\left(u^{\prime },v^{\prime }\right)$ calculated by $H_{i}$ and its corresponding label $\left(u^{\prime \prime },v^{\prime \prime }\right)$ recorded by IMU. The distances between all $\left(u^{\prime },v^{\prime }\right)$ and $\left(u^{\prime \prime },v^{\prime \prime }\right)$ are mapped to a normal distribution to find $\mu_{i}$ and $\sigma_{i}$ of $E_{i}$.

By mapping all pixels of $C_{i}$ to the ground, we can obtain the coverage $R_{i}$ of $C_{i}$. The next task is to find the intersection $R_{i}\cap R_{j}$ for each pair of $C_{i}$ and $C_{j}$. The shape of $R_{i}$ is close to a polygon. Therefore, the overlapping area $R_{i}\cap R_{j}$ is also close to a polygon. The related backgrounds can be found in [33,34].

**Figure 3:**
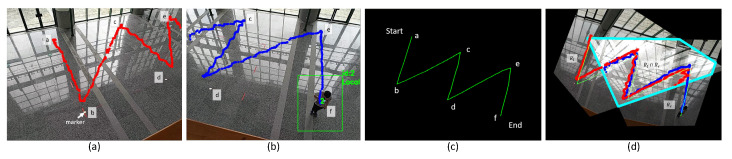
(**a**) Trace found by $C_{1}$. (**b**) Trace found by $C_{2}$. (**c**) IMU trajectory partitioned by markers. (**d**) Overlapping region $R_{1}\cap R_{2}$.

Figure 3 shows an example. Figure 3a,b are views taken by $C_{1}$ and $C_{2}$ at the same time. There are six tiny markers on the ground (we do not require cameras to recognize these markers). Through human detection, trajectories of people are obtained. Meanwhile, wearable sensors also derive their trajectories. Figure 3c shows the rescaled IMU trajectories. Finally, by $H_{1}$ and $H_{2}$, the overlapping region $R_{1}\cap R_{2}$ is obtained in Figure 3d.

### 3.3. Local Object Detection and Tracking

Each camera $C_{i}$ needs to detect and track human objects locally. Since this task has been extensively researched, we will only discuss how we use existing tools to solve this problem. First, for each $F_{t}^{C_{i}}$, it is sent to YOLOv3 [2], a real-time deep learning model for object detection, to retrieve a set of bounding boxes representing detected human objects. We have also tried skeleton models by OpenPose [3], but since the detection time is longer, we adopt YOLO in the rest of the discussion.

Second, we need to determine if a human object detected in $F_{t}^{C_{i}}$ has also appeared in $F_{t-1}^{C_{i}}$. This is achieved by Simple Online and Realtime Tracking (SORT) [13], a tracking algorithm based on the Kalman filter. The outputs are a detected ID set $O_{t}^{C_{i}}=\left\{ID_{x}^{C_{i}},ID_{y}^{C_{i}},...\right\}$ corresponding to all bounding boxes and a miss ID set $M_{t}^{C_{i}}=\left\{ID_{a}^{C_{i}},ID_{b}^{C_{i}},...\right\}$. We call $ID_{x}^{C_{i}}\in O_{t}^{C_{i}}$ the *local* ID assigned to object *x* at time *t* by camera $C_{i}$. If $ID_{x}^{C_{i}}$ appears in both $O_{t}^{C_{i}}$ and $O_{t-1}^{C_{i}}$, object *x* is regarded as the same person. On the other hand, false negative ($x\in O_{t-i}^{C_{i}}$, but *x* is not detected as an object at *t*) and ID switching (*x* receives different IDs at t−1 and *t*) may happen. If an object in $O_{t-i}^{C_{i}}$ disappears in $O_{t}^{C_{i}}$, the corresponding predicted bounding boxes from Kalman filter are included in $M_{t}^{C_{i}}$. In fact, ID switching is not uncommon when YOLO continuously fails to detect an object or network packets are lost continuously, making SORT regard it as a new person after it reappears. It also happens when a detected bounding box drifts far away from its previous location in a new frame. We will discuss how to reduce such confusion later.

### 3.4. Global Object Tracking

**Figure 4:**
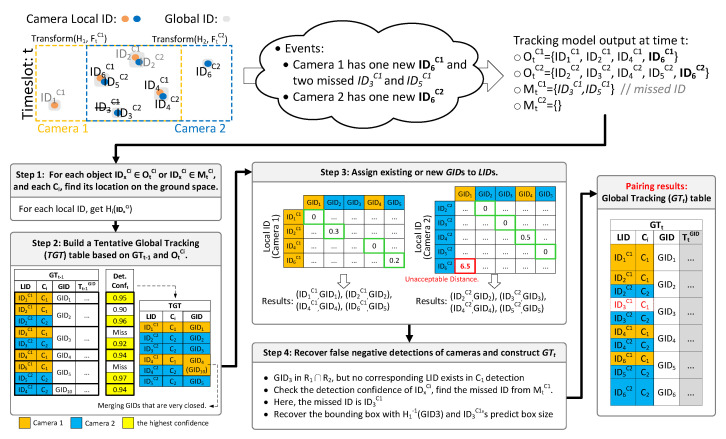
An example of creating $GT_{t}$ from $GT_{t-1}$.

The next objective is to merge all $O_{t}^{C_{i}}$s detected by all cameras under a global domain. Assume that at time t−1 a Global Tracking table $GT_{t-1}$ as shown in Figure 4 is obtained. The contents of $GT_{t-1}$ include: (i) the local ID (LID) assigned to object *x*, (ii) the source camera $C_{i}$ that captures *x*, (iii) the global ID (GID) assigned to *x*, and (iv) the trajectory $T_{t-1}^{GID}$ of *x*. Note that a GID may be associated to multiple LIDs if the object is captured by more than one camera.

We need to construct $GT_{t}$ at *t* from $GT_{t-1}$. There are four tasks:

1. For each object $ID_{x}^{C_{i}}\in O_{t}^{C_{i}}$ or $ID_{x}^{C_{i}}\in M_{t}^{C_{i}}$, and each $C_{i}$, find *x*’s location on the ground space.
2. Build a Tentative Global Tracking table (TGT).
3. Assign existing or new GIDs to LIDs.
4. Recover false negative detections of cameras and construct $GT_{t}$.

The first task can be achieved by $H_{i}$ of $C_{i}$. Here, we regard the central pixel of an object’s bounding box as its location. The corresponding result is denoted as $H_{i}\left(ID_{x}^{C_{i}}\right)$.

The second task is to build TGT based on $GT_{t-1}$ and the new tracking results $O_{t}^{C_{i}}$. TGT has the same three entries as $GT_{t-1}$ (i.e., LID, $C_{i}$, and GID). We copy $GT_{t-1}$ into TGT with three modifications: (i) For each LID in $M_{t}^{C_{i}}$, we exclude it in TGT since the object is not detected at *t*. (ii) Depending on the detection confidence at *t*, for each GID, only the entry with the highest confidence is kept. (iii) If two GIDs at t−1 become very close to each other at *t*, they will be merged into one GID (we keep the earlier comer here). For example, in Figure 4, $ID_{3}^{C_{1}}$ and $ID_{5}^{C_{1}}$ disappear at *t*, so they are removed. Furthermore, $GID_{4}\left(ID_{4}^{C_{1}}\right)$ and $GID_{10}\left(ID_{4}^{C_{2}}\right)$ become too close, the latecomer $GID_{10}$ is merged with the earlier comer $GID_{4}$.

The third task is to assign GIDs to LIDs. First, distance matrices between all GIDs and all LIDs of all cameras are built. Given a $GID_{i}$ and a local $ID_{x}^{C_{j}}$ detected by $C_{j}$, we define $d(GID_{i},ID_{x}^{C_{j}})$ as the Euclidean distance between the location of $GID_{i}$ and $H_{j}\left(ID_{x}^{C_{j}}\right)$ (when $GID_{i}$ and $ID_{x}^{C_{j}}$ fall in the same row in TGT, the distance is 0). Then we run the Hungarian Matching algorithm [35,36] to pair GIDs and LIDs in the distance matrix. Since the matched pairs might encounter a false negative match, we execute a threshold test by checking each matched object $ID_{x}^{C_{i}}$ (with the highest detection confidence) in TGT and the corresponding paired object $ID_{y}^{C_{j}}$. Let $\tilde{d}=d\left(H_{i}\left(ID_{x}^{C_{i}}\right),H_{j}\left(ID_{y}^{C_{j}}\right)\right)$. The confidence that $ID_{x}^{C_{i}}$ and $ID_{y}^{C_{j}}$ belong to the same global object is written as $E_{i}\left(\tilde{d}\right)\cdot E_{j}\left(\tilde{d}\right)$. Intuitively, $E_{i}\left(resp.,E_{j}\right)$ is the confidence that an object at an error distance of $\tilde{d}$ is acceptable. An example is shown in Figure 4. Note that a pair with distance 0 is always matched (so they can be optimized from the Hungarian algorithm). For a LID that is not matched with a GID in the above process, or the remainder, a new GID is assigned to it ($GID_{1}$ and $ID_{6}^{C_{2}}$ is such cases). The final results are a number of (LID,GID) pairs.

In the fourth task, we first recover false negative detections for each $C_{i}$. As we have the intersection region $R_{i}\cap R_{j}$ for each pair of $C_{i}$ and $C_{j}$, if there is a discrepancy such that an $ID_{x}^{C_{i}}$ appears in $R_{i}\cap R_{j}$ but no corresponding $ID_{x}^{C_{j}}$ exists in $C_{j}$’s detection, a miss detection may happen for $C_{j}$. We select the best matched missing LID in $M_{t}^{C_{j}}$, and test its predicted location from SORT against $C_{i}$’s detection $H_{i}\left(ID_{x}^{C_{i}}\right)$. If the test is passed, we can recover the missing bounding box by applying $H_{j}^{-1}$ on $H_{i}\left(ID_{x}^{C_{i}}\right)$ and the predicted box size from $M_{t}^{C_{j}}$, and add it to $C_{j}$’s local detection list $O_{t}^{C_{j}}$. The new bounding box of $C_{j}$ should belong to the same GID of $C_{i}$’s. Finally, we can compile all (LID,GID) pairs to construct the $GT_{t}$ at *t*.

### 3.5. Feature Extraction

In order to pair video objects with devices, we have to extract some common features to compare their similarity. Let $f_{t}^{GID_{i}}$ and $f_{t}^{D_{j}}$ be the features extracted from the video trajectory of $GID_{i}$ and device $D_{j}$, respectively, within a sliding window [t−Δt,t]. We will derive two features, activity type and moving direction. For the activity type of $GID_{i}$, there are four types, 0 for standing, 1 for walking, 2 for turning-left, and 3 for turning-right. To train a Support Vector Machine (SVM), we feed mean, standard deviation, upper and lower quarter, and median absolute deviation of a number of key sequences as inputs, where a time slot is a basic unit of a sliding window:

1. Δdistance: distance between each sampling point in a time slot.
2. Δangle: angle between each sampling point in a time slot.
3. Δaxis: axis between each sampling point in a time slot.

The classification result is denoted as $f_{t}^{GID_{i}}\left[act\right]$. For the activity type of $D_{j}$, it is derived by the following key sequences:

1. Three-axis accelerometer reading.
2. Three-axis magnetometer reading.

In addition, by SVM, the classification result is denoted by $f_{t}^{D_{j}}\left[act\right]$.

**Figure 5:**
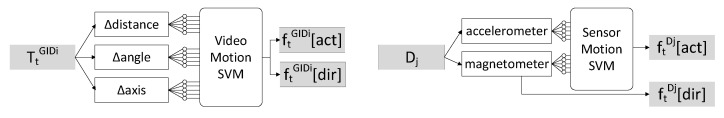
Feature extraction procedure for $GID_{i}$ and $D_{j}$.

The moving direction is a 2D normalized vector in the world space. The moving direction of $GID_{i}$ is denoted by $f_{t}^{GID_{i}}\left[dir\right]$ and is calculated by the starting and ending sampling points in each time slot. The moving direction of $D_{j}$ is denoted by $f_{t}^{D_{j}}$, which is converted directly from the magnetometer. The value is stabilized by linear regression. Figure 5 illustrates the above procedure for video data and sensor data. $GID_{i}$’s trajectory is taken from $T_{t}^{GID_{i}}$ of $GT_{t}$, while the input from device $D_{j}$ is directly taken from its sensors.

### 3.6. Device and Global Object Pairing

By comparing the similarity between $f_{t}^{D_{j}}$ and $f_{t}^{GID_{i}}$, we try to determine if $GID_{i}$ and $D_{j}$ are a pair. We will calculate two matrices: (i) short-term distance matrix $\delta_{t}$ and (ii) long-term weight matrix $\omega_{t}$. These matrices include all GIDs and devices. By considering short- and long-term relations, we try to obtain more stable pairing results.

**Figure 6:**
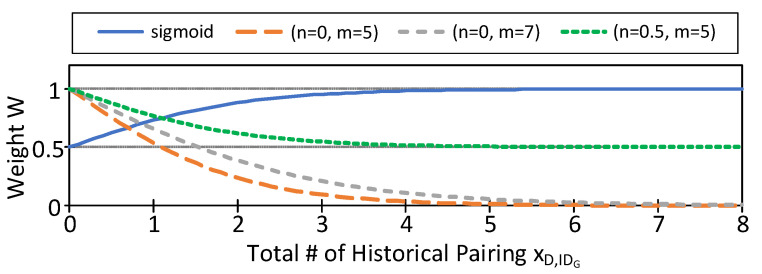
Equation (2) under different parameters.

The matrix $\delta_{t}$ is formed by the distances between all $(f_{t}^{D_{j}}$, $f_{t}^{GID_{i}})$ pairs. Recall vectors $f_{t}^{GID_{i}}\left[act\right]$, $f_{t}^{GID_{i}}\left[dir\right]$, $f_{t}^{D_{j}}\left[act\right]$ and $f_{t}^{D_{j}}\left[dir\right]$. We define short-term distance as

(1) $$\begin{array}{cc} \delta (GID_{i},D_{j})= & (d_{norm}{\left(f_{t}^{GID_{i}}\left[act\right],f_{t}^{D_{j}}\left[act\right]\right)}^{2}\\ \; & +d_{norm}{\left(f_{t}^{GID_{i}}\left[dir\right],f_{t}^{D_{j}}\left[dir\right]\right)}^{2}{)}^{\frac{1}{2}}, \end{array}$$

where $d_{norm}$ is the distance between two vectors by normalizing to the range [0, 1]. We then apply Hungarian matching on matrix $\delta_{t}$ to find the set of pairs denoted by $H(\delta_{t})$. Note that this only reveals the result within the sliding window [t−Δt,t]. The long-term weight matrix $\omega_{t}$ is formed by considering a sequence of short-term results, namely $H\left(\delta_{t-\Delta }\right),H\left(\delta_{t-\Delta +1}\right),...,H\left(\delta_{t}\right)$. For each $\left(GID_{i},D_{j}\right)$ pair, we define long-term distance as

(2) $$\begin{array}{c} \omega_{t}\left(GID_{i},D_{j}\right)=1+\left(1-n\right)-\frac{2(1-n)}{e^{-5cnt(GID_{i},D_{j})/m}}, \end{array}$$

where $cnt(GID_{i},D_{j})$ is the number of times that $\left(GID_{i},D_{j}\right)$ appears in $H(\delta_{t-\Delta })$, $H(\delta_{t-\Delta +1})$, ..., $H(\delta_{t})$. Equation (2) is to simulate an inverse sigmoid function, where *n* is the convergence limit, *m* is the value of $cnt(GID_{i},D_{j})$ to meet *n*, and 5 is a fine-tuned value. Figure 6 shows $\omega_{t}\left(GID_{i},D_{j}\right)$ under different *n* and *m* as opposed to a typical sigmoid function.

**Figure 7:**
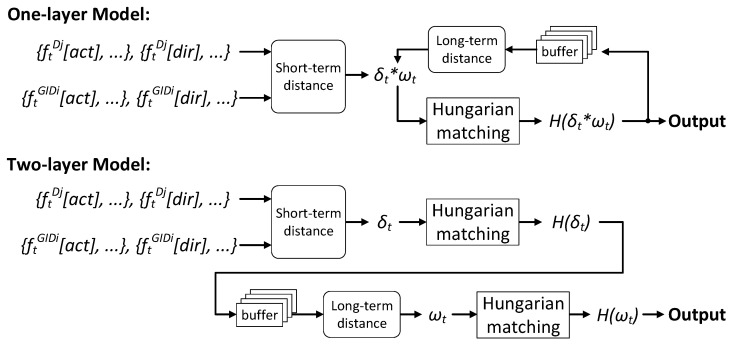
The proposed one-layer and two-layer pairing models.

Based on $\delta_{t}$ and $\omega_{t}$, we propose two pairing models in Figure 7. The one-layer model uses only one Hungarian matching, which takes accumulated $H(\delta_{t}*\omega_{t})$ as inputs, where ∗ means pairwise multiplication. However, we find it to be weak in handling the ID exchange problem during tracking. Therefore, we propose the two-layer model by applying Hungarian matching twice, the first time on $\delta_{t}$ and the second time on $\omega_{t}$. We will validate this claim by experiments.

## 4. Performance Evaluation and Discussion

### 4.1. Experimental Setting

We built a prototype, which consists of two Nokia 8.1 smartphones to simulate IP cameras. We set their height to six meters, view angle to 50 degrees down, and resolution to 600 × 800 pixels. These phones connect to an IEEE 802.11ac access point and stream videos at 20 FPS to our fusion server. Wearable devices are simulated by Android smartphones, which also connect to our fusion server and transmit sensor data at a rate of 50 Hz. The wireless router is ASUS RT-AC86U with a Cortex A53 1.8 GHz dual-core processor and 256 MB RAM. Its claimed rate is 1734 Mbps under 802.11ac and 450 Mbps under 802.11n. Our fusion server has an Intel i7-9750H CPU with six cores, 32 GB RAM, and a RTX 2080 MAX-Q GPU. To speed up the detection speed of YOLOv3-608 (which resizes images to 608 × 608), we added an external RTX 2080 eGPU. Table 1 shows the respective processing time. Processing one frame takes 0.081 s, which approximates to 12 FPS in real-time. This value is calculated from a 5-min clip with processing around 6000 frames. Table 2 shows the activity recognition results of our SVM model, where V-SVM is video motion SVM, and S-SVM is sensor motion SVM. It is trained by a 12-min video by taking 80% data for training and 20% for testing. The accuracies are 97.5% and 99.6% for classifying the four motions from video and sensor data, respectively. The precision and recall are around 95% to 98% for four types of motions. The results show that SVM models are reliable for obtaining motion features.

**Figure 8:**
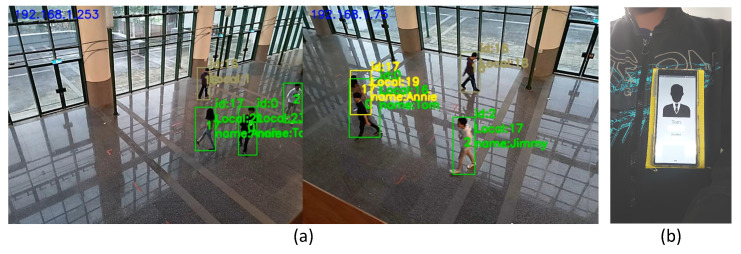
(**a**) Experiment scene. (**b**) Smartphone setup on chest.

**Figure 9:**
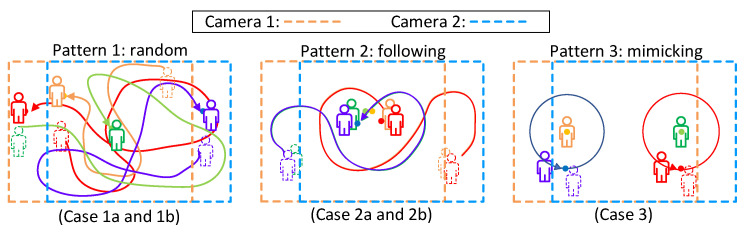
Mobility patterns for experiments.

Figure 8 shows our experimental scenario. We invited four testers to walk under the cameras. Three of them wear a smartphone on their chests, as shown in Figure 8b. The last one is a guest without any device. In Figure 8a, an identified person will be tagged as a green bounding box with its corresponding profile. The yellow bounding box indicates the result of recovered missed detection, which will be discussed in Section 4.3. We designed three mobility patterns, as shown in Figure 9. Pattern 1 is a random walk to observe tracking ability when people are walking freely inside the area. We generated two trials. Case 1a and 1b are two different random trajectories. Pattern 2 is following with multiple interleaving, which may cause an ID switch due to SORT [13], because SORT does not consider the target’s visual features on frames. We also generated two trials (2a and 2b) for pattern 2 to observe the ID switch issue. Our system can solve this issue by recovering the lost bounding box. Pattern 3 is trajectory mimicking to simulate that people have similar moving patterns. Since the moving patterns are similar, we generated one trial (case 3) for pattern 3. In each case, video clips and sensor data were collected for 5 min.

Our evaluations are based on four metrics: (i) PITA (Person Identification and Tracking Accuracy), (ii) IDP (Identification Precision), (iii) IDR (Identification Recall) and (iv) IDF1 (Identification F-score 1).

(3) $$\begin{array}{cc} \mathrm{PITA} & =1-\frac{\sum_{t}^{N}{\left(ObjMiss+IncPair\right)}_{t}}{\sum_{t}^{N}{\left(truth\#\right)}_{t}} \end{array}$$

(4) $$\begin{array}{cc} \mathrm{IDP} & =\frac{\mathrm{TP}}{\mathrm{TP}+\mathrm{FP}} \end{array}$$

(5) $$\begin{array}{cc} \mathrm{IDR} & =\frac{\mathrm{TP}}{\mathrm{TP}+\mathrm{FN}} \end{array}$$

(6) $$\begin{array}{cc} \mathrm{IDF}1 & =2\frac{\mathrm{IDP}*\mathrm{IDR}}{\mathrm{IDP}+\mathrm{IDR}} \end{array}$$

In the above, ObjMiss means missing human object detection (including false positive and negative), and IncPair means incorrect pairing of devices and human objects. TP is the number of true positive pairs, FP is the number of false positive pairs, and FN is the number of false negative pairs.

### 4.2. Pairing Accuracy

**Figure 10:**
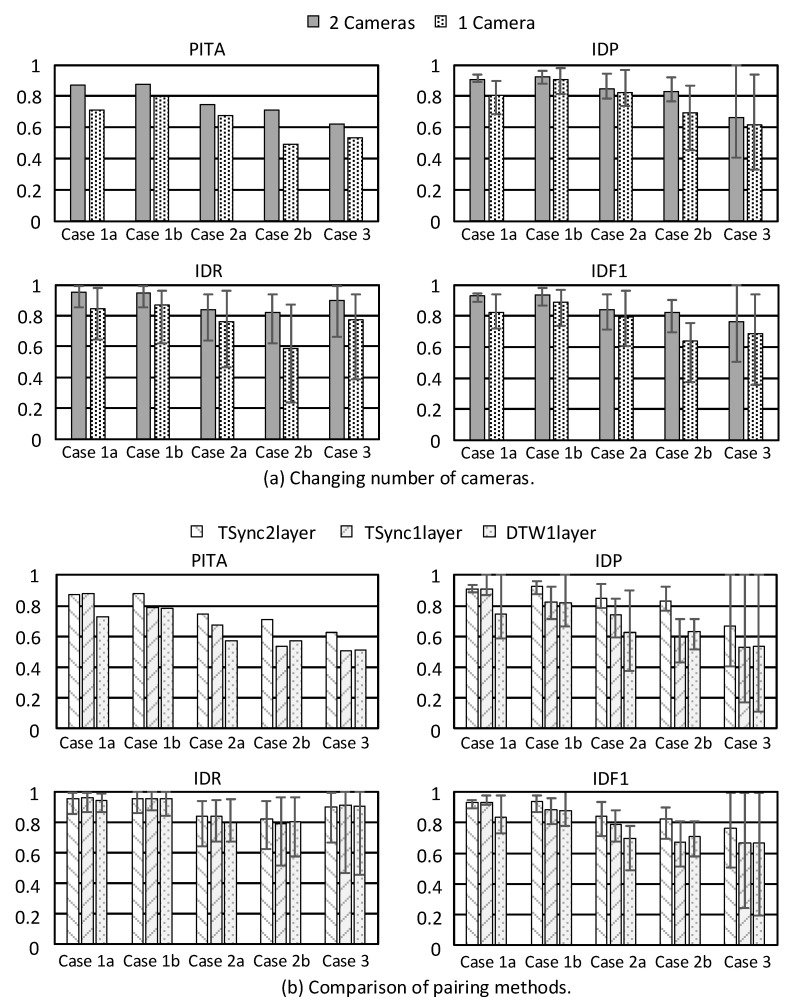
Pairing accuracy.

First, we compare single-camera versus multi-camera cases with the two-layer method. Figure 10a shows PITA, IDP, IDR and IDF1 under different mobility patterns. PITA is the accuracy of a whole video clip and sensor data. The value of IDP, IDR and IDF1 is calculated for each person. When more than two people appear in the scene, several pairing results are obtained. We point out the maximum and minimum values in Figure 10 to examine the robustness of our model. Using multiple cameras gives higher accuracy in all cases because it provides more view angles on the environment, thus improving vision recognition capability. On average, accuracy is increased by 10%. The difference between the maximum and minimum value of IDP, IDR, and IDF1 under two cameras is lower than one camera, which means using multiple cameras is more robust.

Next, we compare the two proposed pairing methods. Here, 1L and 2L mean 1-layer and 2-layer methods, respectively. TSync means using time synchronization; otherwise, Dynamic Time Warping (DTW [37]) will be applied. For Equation (2), *n* is set to 0.9 for DTW1L and TSync1L, and *n* is set to 0 for TSync2L. The number *m* of historical records is set to 10, and the fusion frequency is 2 Hz. Figure 10b shows the results using different pairing methods, where there are always two cameras. We can see that TSync2L performs the best in most cases. For mobility pattern 2, false negative detection occurs due to obstacles. Therefore, TSync1L and DTW1L have much lower accuracy than TSync2L. On average, TSync2L leads by 10% in PITA and IDF1. Considering the difference between the maximum and minimum value, TSync2L is the most robust, and TSync1L outperforms DTW1L.

When DTW is applied, short-term data misalignment may be recoverable. However, it is hard to recover longer misalignment. Therefore, clock synchronization is more important when there are more devices and cameras.

When investigating object tracking, it is common to use an F-score of 1 for comparison. Table 3 shows the detail IDF1 of each person in each case. When the system can not determine the GID of an object, we set it as “unknown”. This would make IDF1 decrease significantly. From Table 3, we can see that IDF1 is greater than 90% under pattern 1. In pattern 2, there are more detection failures, ID switches/exchanges, and occlusions for the computer vision task. Even so, our system can still track people with IDF1 of 80%. Unfortunately, pattern 3 is a challenge to our system because people have similar movements. Our current movement features are unable to distinguish such cases. A possible solution is to explore more detailed features, such as skeleton data, which can be a direction of future work.

### 4.3. False Negative Recovery Capability

**Figure 11:**
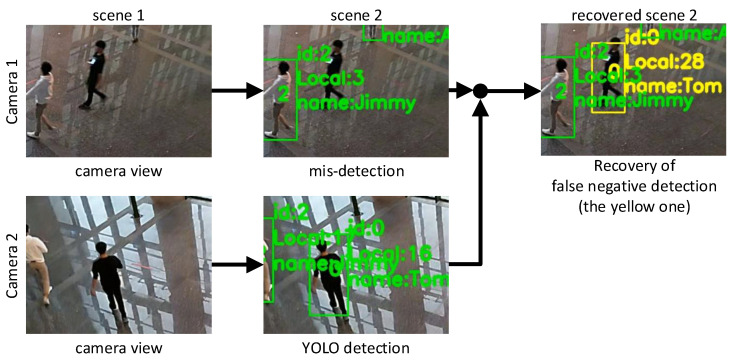
False negative recovery scenario.

False negative detection is inevitable in image object detection. As shown earlier, this may be recovered by our system in camera intersection regions. In order to observe the recovery capability, we collected 1886 pieces of data in the overlapping area $R_{1}\cap R_{2}$ when a person moves in. Ideally, both cameras $C_{1}$ and $C_{2}$ should be able to detect and track them. We treat the tracking results of $C_{1}$ as ground truth. When solving the device–object pairing problem, we assume that $C_{1}$ cannot detect the person, and then we use the information from $C_{2}$ to recover the missed detection of $C_{1}$. The recovered detection is regarded as a new bounding box of $C_{1}$, and then we compare these new bounding boxes against those detected by the same $C_{1}$ through YOLO. Figure 11 shows a scenario. A person in black clothes is in the overlapping area $R_{1}\cap R_{2}$. Although both $C_{1}$ and $C_{2}$ may detect the person, we purposefully remove $C_{1}$’s detection. During the data fusion procedure, we use the information from $C_{2}$ to recover the lost bounding box on $C_{1}$. Then the predicted results for $C_{1}$ are compared against the ground truth (i.e., the one detected by YOLO). The outcomes are measured by Intersection over Union (IoU), IoU=(AreaofOverlap)/(AreaofUnion), of these two bounding boxes. An IoU value closer to 1 indicates a better recovery effect.

**Figure 12:**
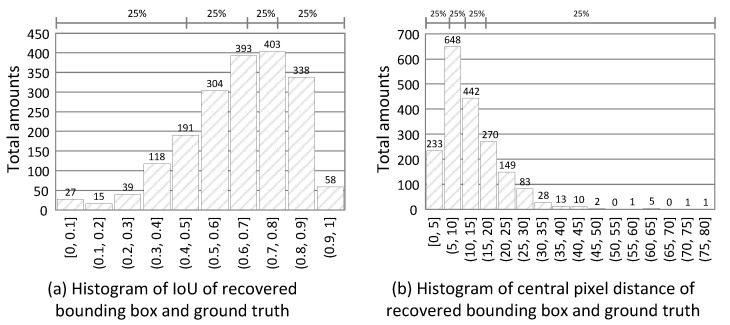
Evaluation results for false negative recovery.

Figure 12a shows the histogram of IoU of these 1886 tests. We see that the top three IoU ranges fall in the (0.6, 0.9] interval, and 50% of results are in the (0.6, 1] interval. Figure 12b shows the histogram of the central pixel distance of two bounding boxes. We see that over 75% of the distances fall in the (0, 15] interval. Even though the view angles of $C_{1}$ and $C_{2}$ are different, most of our predicted bounding boxes by $C_{2}$ can achieve an IoU above 0.7 and a pixel distance of less than 20. On average, the mean IoU is 0.64, and the mean distance is 12 pixels. For surveillance applications, such driftings are acceptable, and thus the solving device–object pairing problem helps us to identify and track people or things from different angles and visualize their sensor data easily.

## 5. Conclusions

The device–object pairing problem arises as an essential issue in the IoT world when we concurrently track a group of things with both cameras and sensors. Correctly pairing visual objects with their sensor data may enable lots of new applications. For example, in high-value livestock farming, the visual objects can be defined as animals. In an automatic warehouse, we can treat the robots as visual objects. Based on our system, we can interpret the status of visual objects with sensor information. This work proposes a device–object pairing system consisting of multiple cameras and wearable devices. The overlapping area between cameras can be used for detecting identical objects. When a missed detection occurs, we can use the information from another camera to recover the missing bounding box. We design a lightweight human-assisted process to estimate a homography matrix for each camera that transforms each camera pixel to a ground coordination. The procedure can be finished within a few minutes. To find the relationship between wearable devices and visual objects, we extract motion features from them and then design one-layer and two-layer algorithms to predict possible pairing. A prototype has been built to test the feasibility of our system under several actual scenarios. It also demonstrates the ability to recover the missing bounding box. Future work may be directed to considering larger scales, which involve a more efficient deployment procedure and an IoT platform for device management. In order to improve device–object pairing results, more features, such as sub-meter indoor localization or skeleton recognition, can be taken into consideration.

## Figures and Tables

**Table 1:** Processing time of major components.

	YOLOv3 (2 Cameras)	SORT	GID Tracking	Device Pairing	Overall
Average Time Consumption (s)	0.058	0.003	0.014	0.002	0.081

**Table 2:** Evaluation of SVM activity recognition models.

	Train#	Test#	Accuracy					
V-SVM	11,020	2,756	0.975					
S-SVM	143,164	35,792	0.996					
	**Stop**		**Straight**		**Turn Left**		**Turn Right**	
	**Precision**	**Recall**	**Precision**	**Recall**	**Precision**	**Recall**	**Precision**	**Recall**
V-SVM	0.997	0.997	0.948	0.981	0.964	0.981	0.991	0.968
S-SVM	0.989	0.999	0.994	0.989	0.999	0.996	0.999	0.996

**Table 3:** Comparison of Per-person IDF1.

	Case 1a	Case 1b	Case 2a	Case 2b	Case 3
User 1	0.937	0.972	0.932	0.88	0.992
User 2	0.915	0.910	0.857	0.855	0.867
User 3	0.940	0.975	0.837	0.853	0.557
Unknown	0.927	0.882	0.725	0.71	0.508

## Data Availability

Not applicable.

## Abbreviations

The following abbreviations are used in this manuscript:

YOLO	You Only Look Once
RMPE	Regional Multi-person Pose Estimation
FPS	Frames Per Second
MOT	Multiple Object Tracking
RSSI	Received Signal Strength Indicator
IMU	Inertial Measurement Unit
JSON	JavaScript Object Notation
SORT	Simple Online and Realtime Tracking
GT	Global Tracking table
LID	Local ID
GID	Global ID
TGT	Tentative Global Tracking table
SVM	Support Vector Machine
V-SVM	video motion SVM
S-SVM	sensor motion SVM
PITA	Person Identification and Tracking Accuracy
IDP	Identification Precision
IDR	Identification Recall
IDF1	Identification F-score 1
TSync	Time Synchronization
DTW	Dynamic Time Warping
IoU	Intersection over Union
